# All-Optical Fiber Hanbury Brown & Twiss Interferometer to study 1300 nm single photon emission of a metamorphic InAs Quantum Dot

**DOI:** 10.1038/srep27214

**Published:** 2016-06-03

**Authors:** G. Muñoz-Matutano, D. Barrera, C.R. Fernández-Pousa, R. Chulia-Jordan, L. Seravalli, G. Trevisi, P. Frigeri, S. Sales, J. Martínez-Pastor

**Affiliations:** 1 ITEAM Research Institute, Universidad Politécnica de Valencia, C/Camino de Vera s/n, E-46022, Valencia, Spain; 2 Instituto de Ciencia de los Materiales, Universitat de València, PO Box 22085, E-46071 Valencia, Spain; 3 Departamento de Ingeniería de Comunicaciones, Universidad Miguel Hernández, Avenida Universidad s/n, E-03202 Elche, Spain; 4 CNR-IMEM Institute, Parco delle Scienze 37a, I-43100 Parma, Italy

## Abstract

New optical fiber based spectroscopic tools open the possibility to develop more robust and efficient characterization experiments. Spectral filtering and light reflection have been used to produce compact and versatile fiber based optical cavities and sensors. Moreover, these technologies would be also suitable to study N-photon correlations, where high collection efficiency and frequency tunability is desirable. We demonstrated single photon emission of a single quantum dot emitting at 1300 nm, using a Fiber Bragg Grating for wavelength filtering and InGaAs Avalanche Photodiodes operated in Geiger mode for single photon detection. As we do not observe any significant fine structure splitting for the neutral exciton transition within our spectral resolution (46 μeV), metamorphic QD single photon emission studied with our all-fiber Hanbury Brown & Twiss interferometer could lead to a more efficient analysis of entangled photon sources at telecom wavelength. This all-optical fiber scheme opens the door to new first and second order interferometers to study photon indistinguishability, entangled photon and photon cross correlation in the more interesting telecom wavelengths.

New emerging quantum optics technologies are mostly influenced by the possibility to design realistic proposals to implement and control quantum correlations between photons^1^. Single photon and entangled photon emission has been demonstrated by different techniques and systems^2^, as for example by non-linear processes (parametric down conversion or four wave mixing) or by two level systems (atoms, molecules, Quantum Dots, single impurities or Nitrogen Vacancies in Diamond). However, an entangled photon source must fulfill several requirements for its use in quantum applications^3^: deterministic generation of entangled photons, high fidelity to the Bell state, high photon indistinguishability, and high efficiency. Although non-linear processes generate entangled photons at room temperature, two-level systems offer the opportunity to build a deterministic device, i.e., a system where entangled photons are emitted on demand by an external control (laser pulse or electrical signal). Furthermore, it is interesting to generate entangled photon emission compatible with optical fiber technologies, thus it is necessary to tune the optical emission to the second and third optical telecommunication windows (1300 and 1550 nm).

In this regard, single self-assembled Quantum Dots (SAQDs) are well known solid-state semiconductor nanostructures that offer key advantages as single or entangled photon emitters fabricated on a GaAs substrate. SAQDs show confinement in all dimensions, leading to a 0-dimensional density of states similar to single atoms. The optical emission in the biexciton to neutral exciton cascade has been proposed as a deterministic polarization entangled photon source^4^. Along the last decade, single SAQDs have been used to develop single^5^ and entangled photon emitting diodes as sources of high fidelity Bell states^6^. Single charge states have been controlled in order to manipulate hole spins with very large decoherence times^7^, which is a desirable property in the development of future quantum computing devices. High values of photon indistinguishability have also been obtained in two photon excitation schemes, related to large coherence times^8^. However, most of these milestones were demonstrated mainly by using SAQDs whose optical emission lies on the first telecommunication window (850–980 nm).

Among the possibilities offered by SAQDs engineering, the QD confinement potential can be controlled by the design parameters and the growth conditions, leading to a direct control of electronic states and optical properties, as for example to redshift the photon emission towards longer wavelengths (1300 or 1550 nm). However, in some cases the change in the design of the QD based structure and the growth techniques necessary to achieve such a wavelength tuning can deteriorate the optical quality: reduction of coherence times, increase of the homogeneous linewidth and spectral diffusion effects by the presence of fluctuating charges in the QD surroundings. These factors will invariably induce a reduction of fidelity and photon indistinguishability. At the same time, Fine Structure Splitting (FSS) from electron and hole exchange interaction usually increases with the QD size^9^, hence reducing the possibility to generate entangled photons.

From a technological point of view, conventional Si detection technology is not useful for telecom wavelengths, as the detector efficiency drops dramatically beyond 1000 nm and InGaAs photodetectors are routinely used in photonic devices instead those based on Si, despite their higher noise characteristics. The use of those infrared detectors introduces a strong limitation in the experimental determination of single photon emission, particularly when the Signal to Noise Ratio (SNR) decreases towards unity, even if the detection scheme is enhanced by use of either gated InGaAs Avalanche Photodiodes (APDs) or changing InGaAs by superconductor based photodetectors^2^,^10^. Single photon emission at 1300–1500 nm under both pulsed^11^ and continuous wave^12^,^13^ excitations, and entangled photon emission at 1300 nm^14^, have been demonstrated on SAQDs by using the aforementioned photodetectors and the appropriate experimental set-ups, or by detection strategies based on frequency upconversion^15^.

In this letter, we propose a new all-optical fiber Hanbury Brown & Twiss (HBT) interferometer containing a tunable Fiber Bragg Grating (FBG) optical filtering stage coupled to Geiger mode InGaAs Avalanche Photodiodes (APDs) for single photon detection (Fig. 1). The use of the FBG reduces the complexity and cost of the experimental set-up, avoiding the use of monochromators, free-space optics filters, lenses and collimators, and hence increasing the collection efficiency by more than one order of magnitude^16^. The increase in photon collection efficiency translates into an increment of the SNR when light is detected by means of InGaAs APDs, and thus it will allow the identification of single-photon emission with APDs operated in the conventional Geiger mode. Our all-optical fiber HBT interferometer scheme does not require neither liquid helium operational temperatures, as for the case of superconductor photodetectors, nor synchronization, as in gated InGaAs APDs, and thus offers a compact detection set-up design using the same optical fiber that collects photons from SAQDs. Such a detection scheme would be the basis for a direct way to connect single photons into more complex telecom architectures and/or photonics chips. With the proposed all-optical fiber HBT interferometer we have identified single photon emission after noise subtraction from neutral exciton recombination in metamorphic InAs SAQDs emitting at the second telecommunication window (1300 nm), under cw and pulsed excitation conditions. We have labelled excitonic complexes in our InAs SAQDs by means of micro-Photoluminescence (μ-PL), polarization-selective μ-PL and time resolved μ-PL (μ-TRPL) experiments. Our excitonic assignation corresponds to a large SAQD with an excess of holes in its initial configuration. We identified all fine structure from all excitonic transitions of the QD, where neutral exciton transition does not show any significant FSS within our spectral resolution (46 μeV). Our spectroscopic data is in agreement with the structural information of the SAQD epitaxial growth, and hence our metamorphic SAQD should represent an alternative source for polarization entangled photon emission.

## Results

### Excitonic Labelling of single Quantum Dot Transitions

Typical μ-PL spectra of our large size SAQDs are composed by a relatively high number of optical transitions, which originate from the multiple carrier configurations through the different QD levels. The number of available confined states increases as the QD becomes wider and hence charge carriers can recombine through multiple state configurations. In order to identify and label all these transitions, we performed power excitation, time resolved and polarization resolved μ-PL experiments. Figure 2a shows the μ-PL evolution as a function of the excitation power with the laser resonantly tuned to our Heavy Hole (HH)- WL transition (1.31 eV = 940 nm), exciting the sample in CW mode. This excitation wavelength is selected in order to significantly reduce the spectral diffusion (SD) effect related to random perturbations of the SAQD by a fluctuating charged environment, so that photogenerated carriers are practically independent of the extrinsic carrier dynamics in the GaAs barriers (see more details in the supplementary information). This strategy reduces the extrinsic broadening of the QD optical transitions, enabling their separation and classification. We have indicated in Fig. 2 the most probable labeling for the observed excitonic species, including exchange singlet, doublet and triplet states in positive quartons, hot positive trion and single positive charged biexciton, based on the analysis presented below.

The integrated intensity versus power excitation (Inset in Fig. 2a) provides information about the excitonic or biexcitonic nature of the observed optical transitions, as it is needed an average of one/two adsorbed photons to generate the excitonic/biexcitonic quasiparticle. Table 1 lists all slopes for the power dependence of every transition. The low power excitation spectrum is dominated by the neutral exciton (X^0^; one electron and one hole in conduction and valence band S-levels, repectively, i.e. **[1S**_**e**_**1S**_**h**_]), with a slope of 0.98. At an emission energy higher than X^0^, i.e. with negative binding energy, we identify a positive Trion (X^+1^; **[1S**_**e**_**2S**_**h**_]) and a doubly positive charged exciton, a positive Quarton (X^+2^; one electron and two holes in conduction and valence band S-levels, repectively, and one extra hole in the valence band P-level, i.e. **[1S**_**e**_**2S**_**h**_**1P**_**h**_]). X^+2^ recombination is composed by single unpolarized transition (X_Un_^+2^) at a slight lower energy than X^+1^, and two doublets at lower energy (X_D1_^+2^ and X_D2_^+2^), as it has usually found in literature^17^,^18^. The slopes of X^+1^ and X^+2^ transitions are larger than the one of X^0^ (Table 1), as extra carriers are involved in their recombination paths^19^. The second doublet state of the X^+2^ transition (X_D2_^+2^) appears at −3.3 meV with respect to the unpolarized recombination^20^. On the opposite energy side relative to X^0^, and hence with positive binding energy, we identified the neutral biexciton emission (XX^0^; **[2S**_**e**_**2S**_**h**_]), with slope ≈1.68, and a single positive charged biexciton (XX^+1^; **[2S**_**e**_**2S**_**h**_**1P**_**h**_]), with slope ≈1.9. XX^+1^ recombination is produced between electrons and holes in S-levels (S_e_-S_h_) populating the hot positive trion state (X^+1*^; **[1S**_**e**_**1S**_**h**_**1P**_**h**_]). As recently demonstrated in refs 21 and 22, the X^+1*^ transition is composed by singlet and triplet states, whose degeneracy is broken by the hole-hole exchange interaction, whereas the triplet degeneracy is broken by electron and hole exchange interaction. The complete description of the X^+1*^ state diagram, containing all exchange effects is presented in the supplementary information. In Fig. 2a we found that the X^+1*^ triplet state emission is characterized by a slope ≈1.5 for the power dependence of the μ-PL integrated intensity that is smaller than that of XX^+1^, but slightly larger than the one of X^+1^. Finally, we observe a biexcitonic transition with a slope = 1.9, matching the low energy sideband of the X^0^ transition. We were not able to extract enough information to propose a clear excitonic assignation, although here we tentatively assign to the singlet state transition of the hot positive trion X^+1*^.

On the basis of our previous experience in the study of selectively charged quantum dots^23^,^19^, the above described SAQD would represent a dynamical situation with an excess of holes in its initial configuration^22^. We attribute such an excess of holes to either the SAQD size or to the excitation conditions. Big-sized SAQDs are expected to be more exposed to the effect of surface charges in the QD interface, as it has been found for GaAs droplet epitaxy QDs^24^. At the same time, these big-sized QDs show a large number of very close hole states as compared to small QDs, and hence it is reasonable to assume that the probability to feed positively charged excitons can be enhanced under specific excitation conditions. In a previous work we have shown that positive excitonic complexes may be promoted when the laser excitation is resonant to the HH-WL state, observed for smaller InAs SAQDs samples^23^. Furthermore, we recorded power dependent μ-PL evolution with excitation above GaAs barrier (780 nm) (see supplementary information). Using this excitation wavelength, neutral and negative transitions dominates the μ-PL spectrum, as GaAs barrier absorption produces extra single and correlated carrier diffusion and injection feeding channels^25^,^26^. In conclusion, our hypothesis of a positively charged QDs seems reasonable as structural information, excitation conditions, and μ-PL in different excitation conditions support it.

Figure 2b shows μ-TRPL measurements under an intermediate excitation power (560 nW) and the excitation wavelength tuned to 780 nm. We were able to record the μ-PL transients for XX^+1^_T_, XX^0^, X^−1^, X^0^, X_Un_^+2^ and X^+1^ optical transitions, but not for the other transitions, as their μ-PL signal intensity under pulsed excitation was below the noise level. Table 1 lists the time constants obtained in the best fit (see supplementary information for fitting procedure). All transitions exhibit large rise times (≥0.6 ns), which is attributed to the use of an intermediate excitation power under pulsed conditions. In a previous study we have shown that rise time increases as a function of the power excitation^16^ due to the multi-exciton cascade recombination^27^. Hence, single excitonic transitions must wait until this cascade is completed. Consistently with this observation, we have found a slightly larger rise time (≈0.79 ns) for X^0^ transition, that coincides with the decay time for XX^0^ (0.73 ns), thus indicating that the recombination of X^0^ must wait for the radiative decay of XX^0^. These data reinforce our previous excitonic labeling. As we found in previous μ-TRPL measurements in a similar SAQD^16^, our best fit returns very similar decay times for the recombination of neutral exciton and biexciton states (Table 1), which was attributed to the weak confinement regime compatible with these big sized SAQDs^28^,^29^.

### Polarization resolved spectra and Fine Structure Splitting

Figure 3 shows the contour plots of polarization-selective μ-PL spectra acquired for the most important optical transitions. In the majority of the optical transitions we observe that the μ-PL peak energy is independent of the collection polarization (Fig. 3a), except in the case of X^+1*^ and XX^+1^ transitions, which will be discussed below. Within our experimental spectral resolution (≈46 μeV), we do not observe any significant FSS for X^0^ and XX^0^ optical transitions. The mean linewidth measured in our SAQD is about 100 μeV, which is slightly larger than those measured in standard SAQDs emitting at the first telecom window (≈20–80 μeV^23^,^30^,^31^). Hence, if the FSS of X^0^ state is small enough, FSS would be blurred by a broadening that is mainly due to spectral diffusion.

The FSS for the X^2+^ transition is composed of an unpolarized line and two fine-structure split doublets (Fig. 2a), each one composed of two fully polarized lines^20^. X^2+^ spectral features are separated by an energy much smaller (3.5 meV in ref. 20 or 3.1 meV here, as observed in Fig. 2a) than the case of the X^2−^ transition (10.1 meV in ref. 20). The FSS of the X^2+^ polarized doublets is expected to be in the order of the X^0^ FSS and much smaller than that of X^2−^. With our spectral resolution, we do not appreciate any FSS signature for X^2+^ transitions, as also happens for X^0^. It should be noticed that it has been previously shown that a reduction of strain in InAs QDs causes a decrease of the FSS^32^,^33^. As in these metamorphic InAs/InGaAs QDs the mismatch between QDs and confining layers is 6.34%, below the 7.16% value of pseudomorphic InAs/GaAs QDs^34^, this strain effect could also explain the low FSS supposed here for both X^0^ and X^+2^ species. However, transitions labelled as X^+1*^ and XX^+1^ exhibit opposite shifts with polarization-selective detection (zoom in Fig. 3b and profile in Fig. 3c), and a clear FSS ≈160 μeV is measured. The recombination of hot trions (X^−1*^ and X^+1*^) are expected to exhibit a FSS of the same nature as that observed for X^0^, but it is characterized by a larger magnitude (see supplementary information for a complete description of the positive hot trion state diagram and recombination)^21^,^22^,^35^. The origin of such large FSS for the X^+1*^ state, is related to the P-type symmetry of the envelope wavefunction of the excited state hole shell that would enhance the anisotropy of the electron-hole exchange interaction^35^. Following these arguments, we conclude that the FSS value of X^0^ and X^+2^ are well out of our spectral resolution or blurred by energy incertitude due to line broadening effects, whereas the measured FFS of X^+1*^ is intrinsically large, ≈160 μeV.

### Single photon emission characterization

Figure 4a shows the photon autocorrelation coincidence measurement for X^0^ recombination with CW excitation tuned to 940 nm. In order to extract information about the single photon emitter features we analyzed our results following the standard protocol^36^. The HBT measurement returns a coincidence photon counting plot as a function of time delay (C(τ)) that must be normalized following the relation:


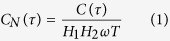


where *H*_*i*_ = *C*_*i*_ + *N*_*i*_ is composed by the sum of the optical signal from QD emission (*C*_*i*_) and dark counts (*N*_*i*_) from detection channel *i* (Start/Stop), ω is the time bin of the correlation board (190 ps) and T is the total integration time (11 hours). We fitted the normalized photon coincidence plot using the expression of the second order correlation function of a two level system, (*g*^2^(*τ*)), convolved by a Gaussian function representing the detector time resolution:


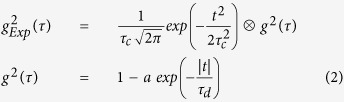


where τ_c_ is the FWHM of the instrumental response (400 ps), τ_d_ is decay time of the transition and 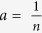, where *n* is the average number of photons in the photon stream. Following this procedure our fitting routine returns *g*^2^(0) = 0.30 ± 0.12. When the photon correlation is measured with small SNR it must be considered the noise effect from dark count coincidences. In our experiment we found 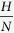 ratios of 1900/10 (start channel) and 1800/560 (stop channel) for id230 and id220 APDs, respectively. In order to take into account the unbalanced effect of noise, we used the following expression^37^:


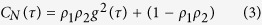


where 
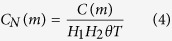
. From our signal and noise measurement ρ_1_ = 0.99 and ρ_2_ = 0.69. Under these detector conditions, an ideal single photon emission, (*g*^2^(0) = 0), would produce dark count correlations of *C*_*N*_(0) = 0.29 ± 0.04, which coincides with the experimental value extracted above by the fitting procedure. Therefore, we conclude that the X^0^ transition of our SAQD is compatible with *g*^2^(0) = 0.01 ± 0.16 after noise subtraction, which produces *n* = 1.01 ± 0.16 average photons.

Figure 4b shows the photon correlation experiment for the same X^0^ transition under pulsed excitation at 780 nm. The process to obtain the information for *g*^2^(0) is similar to that developed above for CW excitation, except that under pulsed excitation the photon correlation counts are distributed periodically along the delay time axis and separated by the laser period. To normalize the coincidence counts now we use the expression:


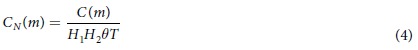


where *m* is an integer value corresponding to the pulse number. *C*(*m*) was obtained performing a multi Lorentzian peak fitting to calculate the integrated area of every m-peak, *H_i_* are defined as before, *θ* is the laser repetition period (13 ns) and *T* is the integration time (8 hours). After this normalization we obtain *C*_*N*_(0) = 0.44 ± 0.05. This result compares well with the theoretical value *C*_*N*_(0) = 0.51 ± 0.04, which is deduced by considering the effect of the dark counts correlation assuming an ideal situation (*g*^2^(0) = 0). Again, the *g*^2^(0) value for the X^0^ transition after noise subtraction is close to the ideal case.

## Discussion

We have presented a complete optical study of a single metamorphic InAs SAQD by means of a novel all-optical fiber intensity interferometer containing a FBG-filtering stage coupled to Geiger mode InGaAs APDs. We analyzed and labeled the most relevant excitonic and biexcitonic transitions and identified a FSS ≈160 μeV associated to the single positive charged hot-trion state (X^+1*^). Contrarily, we did not observe any measurable FSS related to neutral exciton (X^0^) and positive quartons (X^+2^). Such a small FSS for X^0^ is compatible with the nominal low lattice mismatch between these InAs/InGaAs SAQDs and the confining layers (6.34%), which has been proved to cause a decrease of the FSS^32^,^33^. These results could also open the way towards the development of entangled photon sources at 1300–1550 nm telecom windows, as metamorphic QDs have been demostrated to be able to emit beyond 1550 nm^38^.

**Figure 1:**
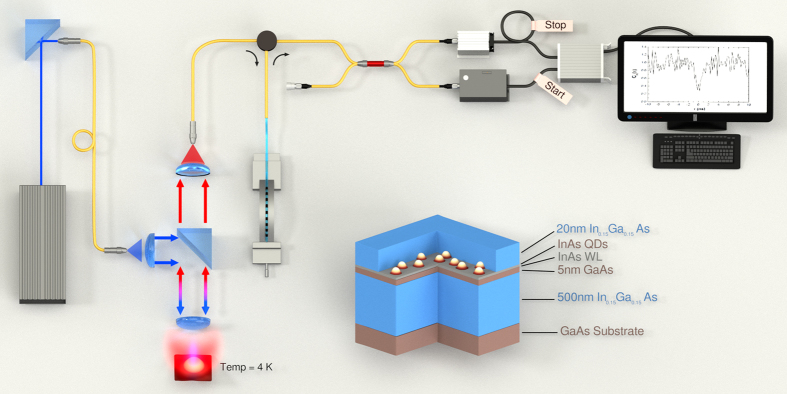
All-optical fiber Hanbury Brown & Twiss interferometry set-up for measuring photon coincidence experiment. From left to right: the laser (pulsed or continuous) is coupled to a single mode fiber. The fiber arrives to the excitation arm of a fiber based confocal microscope, and excite an isolated Quantum Dot at 4 K. Micro-Photoluminescence emission is directed to the collection arm of the microscope and coupled to another single mode fiber. A circulator operating for 1300 nm redistribute the light to the Fiber Bragg Grating installed on a metal strip to change curvature, and hence produce tunable wavelength filtering. The reflected light from the Fiber Bragg Grating arrives to the circulator and is distributed to the entrance of a 50/50 fiber coupler. Each coupler out-put port is attached to InGaAs Geiger mode Avalanche Photodiodes (ID230 at Start and ID220 at Stop channels). Both detector electronic outputs are plugged to a correlator, where stop channel includes a larger path (delay represented as black cable with single loop). Finally, photon coincidence plot is represented in the computer screen. In the same figure is represented the metamorphic QD sample, with brown representing GaAs layers and with blue In_0.15_Ga_0.85_As layers. InAs Wetting Layer and QDs are represented by gray and white colors.

**Figure 2:**
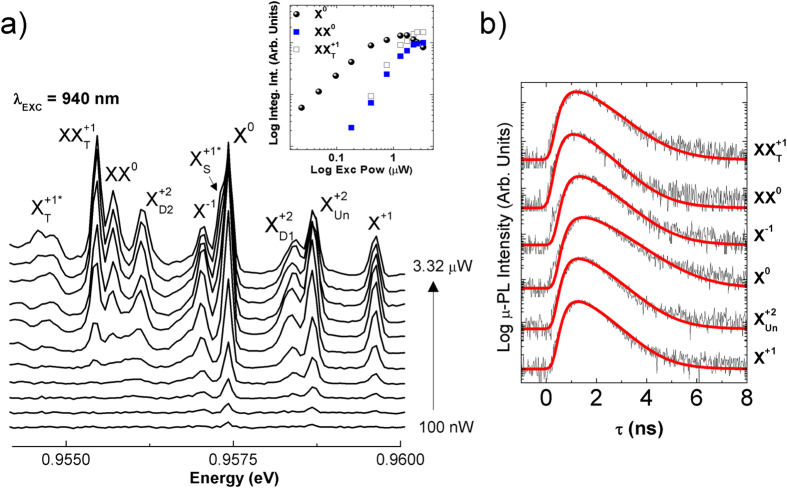
Basic spectroscopic characterization. (**a**) Power dependent micro-Photoluminescence spectra from single metamorphic InAs SAQD emitting ≈1295 nm (≈0.9574 eV) at 4 K, excited with laser resonant to the wetting layer state (940 nm = 1.319 eV) under steady state conditions; (Inset) Integrated intensity versus power excitation in a double logarithmic plot of the fundamental transitions labelled as neutral exciton (X^0^), neutral biexciton (XX^0^) and single positive charged biexciton (XX^+1^). (**b**) Time resolved micro-Photoluminescence of the most intense transitions in figure a) recorded at 4 K under pulsed laser conditions at 780 nm. Red continuous lines represent the best fit to equation [S.1] in the Supplementary information.

**Figure 3:**
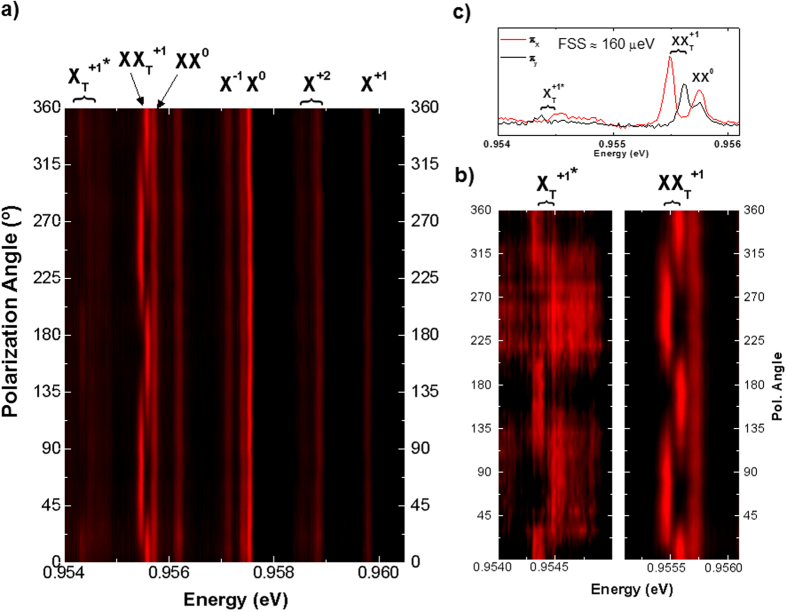
Polarization resolved micro-photoluminescence spectra. (**a**) Polarization-selective μ-PL contour plot of the same SAQD whose optical transitions were shown in Fig. 2 and (**b**) zoom centered at X_T_^+1*^ (Left) and XX_T_^+1^ (Right) transitions; both contour plots show clear opposite shift that follows the FSS versus detection polarization. c) μ-PL spectra with orthogonal linear polarizations (π_x_; red, and π_y_; black) that yield a FSS ≈160 μeV for transitions X_T_^+1*^ and XX_T_^+1^.

**Figure 4:**
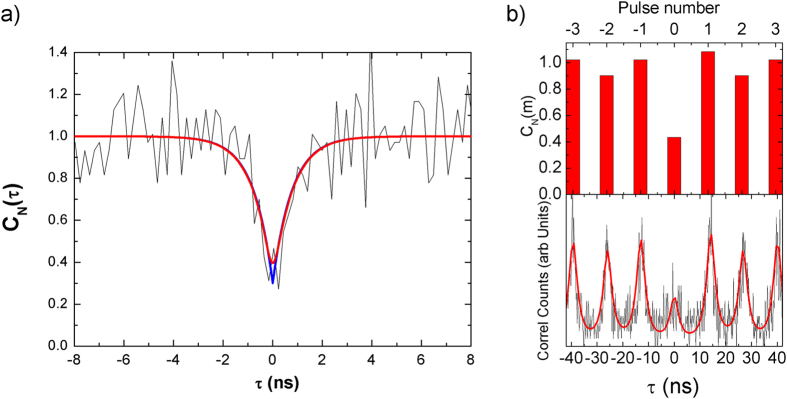
Photon autocorrelation experiments. (**a**) Photon autocorrelation coincidence plot of neutral exciton (X0) optical transition under continuous excitation resonant to the wetting layer state (940 nm = 1.319 eV), at 4 K. Red continuous line correspond to the best fit of the expression^2^, described in the text, which corresponds to a single photon emitter characterized by g^2^(0) = 0.3 ± 0.13. Blue continuous line corresponds to the same fit result without gaussian convolution. (**b**) Photon autocorrelation coincidence plot of the same X^0^ optical transition under pulsed excitation tuned to 780 nm. Red line in bottom panel represents the best Lorentzian fit to the experimental coincidence histogram. Red bars in top panel represent the normalized coincidence count histogram calculated following the process described in text.

Single photon emission of the X^0^ transition at the second telecom window (≈1300 nm) has been characterized, showing that the g^2^(0) value after noise subtraction analysis is close to the ideal case. This represents the first time where single photon emission at 1300 nm has been demonstrated by using InGaAs avalanche photodetectors under Geiger mode operation, which was possible by using a FBG filtering device integrated into the signal collecting optical fiber. The use of FBGs in conjunction with APDs in Geiger mode at 1300 nm is a novelty that offers a relatively simple and compact experimental architecture to study photon indistinguishability and coherence times. As an example of application, it has been recently found new and rich features in the simulation and measurement of frequency filtered N-photon correlation coincidences^39^,^40^. The FBG-filtering stage included in our all-optical fiber photon correlator offers the possibility to measure these bunching and antibunching 2D patterns with an alternative set-up, as FBG filtering produce high collection efficiency, and can be tuned with high frequency/wavelength resolution and high stability. We have previously shown that FBG filtering is suitable to scan single QD transitions^16^. Here we used a broadband FBG in order to match the FBG FWHM with our QD transition linewidths and maximize the photon collection rate, although FBG FWHM can be reduced down to 0.4–3.5 μeV (at 1300 nm)^41^. At the same time, the tuning capabilities of the FBG transmission peaks can be as sensitive as ≈0.4 μeV. These FBG parameters are suitable to filter and scan any conventional QD linewidth, with typical FWHM between 20–100 μeV. Finally, the set-up illustrated in Fig. 1 may be adapted to study N-photon cross correlation architecture with the help of N-FBG filtering devices attached one after another, which represent an easy “plug and play” optical device, with reduced light losses.

In summary, our All-Optical Fiber proposal resembles the conventional procedure used in the analysis of SAQDs emitting below 1000 nm, where Si APDs photodetectors do not exhibit any SNR limitation and are used in the conventional Geiger mode. Our strategy represents a simpler, low cost and efficient tool to connect single/entangled photons into photonic architectures for telecommunications, as for example to develop new N-photon correlation experiments or more compact optical fiber based single and entangled photon devices. Single metamorphic QDs studied with our all-optical fiber based experiment shows single photon emission at 1300 nm with no evidence of neutral exciton Fine Structure Splitting, and hence represents a viable structure design to develop the more interesting deterministic entangled photon source at 1300–1550 nm telecommunication wavelengths.

## Methods

### Sample

In Fig. 1 is shown the schematic of the metamorphic SAQD structure, grown by Molecular Beam Epitaxy. After a 100 nm GaAs buffer, a metamorphic In_0.15_Ga_0.85_As layer of 500 nm was deposited, followed by 5 nm of GaAs. Then a sub-critical coverage of 1.5 ML of InAs was deposited at 490 °C followed by 20 s annealing at the same temperature under As flux; SAQDs were finally capped by a 20 nm thick layer of In_0.15_Ga_0.85_As. Photon emission from these single SAQDs in the long wavelength range has been previously demonstrated^16^,^42^. The use of a metamorphic InGaAs layer allows for the red-shift of the emission wavelength thanks to the reduction of strain of QDs and band discontinuities^43^, whereas the deposition of a sub-critical coverage of InAs followed by thermal annealing allows to obtain very low densities of SAQDs (1 × 10^8^ cm^−2^)^44^. In these samples with In_0.15_Ga_0.85_As metamorphic layers the diameter of the QDs (d_QD_) has been estimated by AFM to be in the 30–45-nm range^44^, which is larger than the diameters of metamorphic QDs grown with the SK method^45^,^34^. Similarly, heights for QDs grown by the sub-critical method are in the 6–9 nm range^44^, larger than those of SK QDs^34^.

### All Optical Fiber Photon Correlation Experiment

Figure 1 also shows a detailed scheme of the all-optical fiber HBT interferometer set up used here to measure single photon coincidences. The QD sample was held in a fiber-based confocal microscope at 4 K to record the single QD optical emission. Single SAQDs were excited by tunable Ti:Saphire laser (Mira 900D) working under either continuous wave (CW) or pulsed excitation (120 ps width). Wavelength selection was carried out by filtering the SAQD emission through a FBG device. The FBG was patterned by means of a CW Frequency-doubled Argon ion laser at 244 nm and a phase mask technique into a photosensitive optical fiber. FBGs are 3 cm long and have an apodization in the modulation of the refractive index that allows reducing the level of the side lobes in the optical spectrum. The FWHM of the different FBGs that we used in the experiments was in the range 150–200 μeV, with a reflectivity of 99.95% of the incident light. After the fabrication, the FBG is completely adhered to the surface of a calibrated thickness metal strip. This structure is placed between two linear stages that curve the metal strip when they approach each other. Changing the curvature of the strip allows tuning the central wavelength by compressing or stretching the optical fiber. In this way, we were able to obtain a complete tuning range of more than 8 nm by using two different FBGs. The detection scheme follows a HBT intensity interferometry, using two InGaAs APDs from idQuantique, each one with a different dark count rate (10 and 560 Hz for ID230 and ID220 models, respectively) and operated in Geiger mode, attached to a TCSPC board (TCC900 from Edinburgh Instruments). Time resolved μ-PL (μ-TRPL) spectra were acquired with the same FBG wavelength filtering and correlating the InGaAs APD output with a laser triggering. The μ-PL spectra were recorded using a conventional detection scheme with 0.5-m focal length grating monochromator (with an optimal resolution of 46 μeV at 1300 nm) and an Andor iDus InGaAs Charged Coupled Device (CCD) detector. For more details about μ-PL and μ-TRPL set-up arrangements see the supplementary information.

## Additional Information

**How to cite this article**: Muñoz-Matutano, G. *et al.* All-optical fiber Hanbury Brown & Twiss interferometer to study 1300 nm single photon emission of a metamorphic InAs Quantum Dot. *Sci. Rep.* **6**, 27214; doi: 10.1038/srep27214 (2016).

## Supplementary Material

Supplementary Information

## Figures and Tables

**Table 1:** Experimental parameters of the excitonic complexes labelled in Fig. 2, obtained from the different experiments: the slope of power linearly dependent micro-Photoluminescence; decay and rise time constants from time resolved micro-Photoluminescence; and Fine Structure Splitting (FSS) from polarization-selective micro-Photoluminescence.

	X^+1^	X^+2^_Un_	X^+2^_D1_	X^0^	X^+1*^_S_?	X^−1^	X^+2^_D2_	XX^0^	XX^+1^_T_	X^+1*^_T_
Slope	1.13	1.13	1.21	0.98	1.95	1.13	1.15	1.63	1.9	1.5
τ_d_ (ns)	0.64	0.68	—	0.87	—	0.72	—	0.73	0.74	—
τ_r_ (ns)	0.62	0.65	—	0.79	—	0.69	—	0.60	0.71	—
FSS (μeV)	—	—	—	—	—	—	—	—	≈160	≈160

## References

[b1] Walmsley I. A. Quantum optics: Science and technology in a new light. Science 348, 525–530 (2015). 25931550 10.1126/science.aab0097

[b2] Eisaman M. D., Fan J., Migdall A. & Polyakov S. Invited review article: single-photon sources and detectors. Rev. Sci. Instrum. 82, 071101 (2011). 21806165 10.1063/1.3610677

[b3] Lu C.-L. & Pan J.-W. Push-button photon entanglement. Nat. Photonics 8, 174–176 (2014).

[b4] Benson O., Santori C., Pelton M. & Yamamoto Y. Regulated and entangled photons from a single quantum dot. Phys. Rev. Lett. 84, 2513–2516 (2000). 11018923 10.1103/PhysRevLett.84.2513

[b5] Yuan Z. *et al.* Electrically driven single-photon source. Science 295, 102–105 (2002). 11743163 10.1126/science.1066790

[b6] Salter C. L. *et al.* An entangled-light-emitting diode. Nature 465, 594–597 (2010). 20520709 10.1038/nature09078

[b7] Brunner D. *et al.* A coherent single-hole spin in a semiconductor. Science 325, 70–72 (2009). 19574387 10.1126/science.1173684

[b8] Müller M., Bounouar S., Jöns K. D., Glässl M. & Michler P. On-demand generation of indistinguishable polarization-entangled photon pairs. Nat. Photonics 8, 234–238 (2014).

[b9] Seguin R. *et al.* Size-dependent fine-structure splitting in self-organized InAs/GaAs quantum dots. Phys. Rev. Lett. 95, 257402 (2005). 16384505 10.1103/PhysRevLett.95.257402

[b10] Hadfield R. H. Single-photon detectors for optical quantum information applications. Nat. Photonics 3, 696–705 (2009).

[b11] Zinoni C. *et al.* Time-resolved and antibunching experiments on single quantum dots at 1300nm. Appl. Phys. Lett. 88, 131102 (2006).

[b12] Liu X. *et al.* Single-photon emission in telecommunication band from an InAs quantum dot grown on InP with molecular-beam epitaxy. Appl. Phys. Lett. 103, 061114 (2013).

[b13] Benyoucef M., Yacob M., Reithmaier J. P., Kettler J. & Michler P. Telecom-wavelength (1.5 μm) single-photon emission from InP-based quantum dots. Appl. Phys. Lett. 103, 162101 (2013).

[b14] Ward M. *et al.* Coherent dynamics of a telecom-wavelength entangled photon source. Nat. Commun. 5, 3316 (2014). 24548976 10.1038/ncomms4316

[b15] Rakher, M. T. *et al.* Quantum transduction of telecommunications-band single photons from a quantum dot by frequency upconversion. Nat. Photonics 4, 786–791 (2010).

[b16] Muñoz-Matutano G. *et al.* Time resolved emission at 1.3 μm of a single InAs quantum dot by using a tunable fibre Bragg grating. Nanotechnology 25, 035204 (2014). 24356330 10.1088/0957-4484/25/3/035204

[b17] Ediger M. *et al.* Peculiar many-body effects revealed in the spectroscopy of highly charged quantum dots. Nature Phys. 3, 774–779 (2007).

[b18] Gerardot B. D. *et al.* Laser spectroscopy of individual quantum dots charged with a single hole. Appl. Phys. Lett. 99, 243112 (2011).

[b19] Gomis-Bresco J. *et al.* Random population model to explain the recombination dynamics in single InAs/GaAs quantum dots under selective optical pumping. New J. Phys. 13, 023022 (2011).

[b20] Ediger M. *et al.* Fine structure of negatively and positively charged excitons in semiconductor quantum dots: electron-hole asymmetry. Phys. Rev. Lett. 98, 036808 (2007). 17358715 10.1103/PhysRevLett.98.036808

[b21] Warming T. *et al.* Hole-hole and electron-hole exchange interactions in single InAs/GaAs quantum dots. Phys. Rev. B 79, 125316 (2009).

[b22] Benny Y. *et al.* Excitation spectroscopy of single quantum dots at tunable positive, neutral, and negative charge states. Phys. Rev. B 86, 085306 (2012).

[b23] Muñoz-Matutano G. *et al.* Selective optical pumping of charged excitons in unintentionally doped InAs quantum dots. Nanotechnology 19, 145711 (2008). 21817777 10.1088/0957-4484/19/14/145711

[b24] Ha N. *et al.* Size-dependent line broadening in the emission spectra of single GaAs quantum dots: Impact of surface charge on spectral diffusion. Phys. Rev. B 92, 075306 (2015).

[b25] Moskalenko E. S. *et al.* Influence of excitation energy on charged exciton formation in self-assembled InAs single quantum dots. Phys. Rev. B 64, 085302 (2001).

[b26] Rivas D. *et al.* Two-color single-photon emission from InAs quantum dots: toward logic information management using quantum light. Nano Lett. 14, 456–463 (2014). 24422533 10.1021/nl403364h

[b27] Dekel E. *et al.* Cascade evolution and radiative recombination of quantum dot multiexcitons studied by time-resolved spectroscopy. Phys. Rev. B 62, 11038 (2000).

[b28] Wimmer M., Nair S. & Shumway J. Biexciton recombination rates in self-assembled quantum dots. Phys. Rev. B 73, 165305 (2006).

[b29] Dalgarno P. A. *et al.* Coulomb interactions in single charged self-assembled quantum dots: Radiative lifetime and recombination energy. Phys. Rev. B 77, 245311 (2008).

[b30] Muñoz-Matutano G. *et al.* Exciton, biexciton and trion recombination dynamics in a single quantum dot under selective optical pumping. Physica E 40, 2100–2103 (2008).

[b31] Birkedal D., Leosson K. & Hvam J. M. Long lived coherence in self-assembled quantum dots. Phys. Rev. Lett. 87, 227401 (2001). 11736426 10.1103/PhysRevLett.87.227401

[b32] Tartakovskii A. *et al.* Effect of thermal annealing and strain engineering on the fine structure of quantum dot excitons. Phys. Rev. B 70, 193303 (2004).

[b33] Goldmann E., Barthel S., Florian M., Schuh K. & Jahnke F. Excitonic fine-structure splitting in telecom-wavelength InAs/GaAs quantum dots: statistical distribution and height-dependence. Appl. Phys. Lett. 103, 242102 (2004).

[b34] Seravalli L., Trevisi G. & Frigeri P. 2D–3D growth transition in metamorphic InAs/InGaAs quantum dots. Cryst. Eng. Comm. 14, 1155–1160 (2012).

[b35] Akimov I., Kavokin K., Hundt A. & Henneberger F. Electron-hole exchange interaction in a negatively charged quantum dot. Phys. Rev. B 71, 075326 (2005).

[b36] Brouri R., Beveratos A., Poizat J. & Grangier P. Photon antibunching in the fluorescence of individual color centers in diamond. Opt. Lett. 25, 1294–1296 (2000). 18066197 10.1364/ol.25.001294

[b37] Mandel L. & Wolf E. Optical Coherence and Quantum Optics. Cambridge University Press (1995).

[b38] Seravalli L., Frigeri P., Trevisi G. & Franchi S. 1.59 μm room temperature emission from metamorphic InAs∕InGaAsInAs∕InGaAs quantum dots grown on GaAs substrates. Appl. Phys. Lett. 92, 213104 (2008).

[b39] Gonzalez-Tudela A., Laussy F. P., Tejedor C., Hartmann M. J. & del Valle E. Two-photon spectra of quantum emitters. New J. Phys. 15, 033036 (2013).

[b40] Peiris M. *et al.* Two-color photon correlations of the light scattered by a quantum dot. Phys. Rev. B 91, 195125 (2015).

[b41] Venghaus L. *Wavelength Filters in Fibre Optics*. Springer Series in Optical Sciences Vol 123 (2006).

[b42] Seravalli L. *et al.* Single quantum dot emission at telecom wavelengths from metamorphic InAs/InGaAs nanostructures grown on GaAs substrates. Appl. Phys. Lett. 98, 173112 (2011).

[b43] Seravalli L. *et al.* Quantum dot strain engineering of InAs∕InGaAsInAs∕InGaAs nanostructures. J. Appl. Phys. 101, 024313 (2007).

[b44] Seravalli L., Trevisi G. & Frigeri P. Design and growth of metamorphic InAs/InGaAs quantum dots for single photon emission in the telecom window. Crys. Eng. Comm. 14, 6833–6838 (2012).

[b45] Seravalli L., Frigeri P., Nasi L., Trevisi G. & Bocchi C. Metamorphic quantum dots: quite different nanostructures. J. Appl. Phys. 108, 064324 (2010).

