# Supplementary material for: All-Optical Fiber Hanbury Brown & Twiss Interferometer to study 1300 nm single photon emission of a metamorphic InAs Quantum Dot
Source: Sci Rep. 2016 Jun 3;6:27214. doi: 10.1038/srep27214 (PMC4891669; doi:10.1038/srep27214)
Supplement: Supplementary Information [file srep27214-s1.pdf]

Supplementary information

All-Optical Fiber Hanbury Brown & Twiss Interferometer to study 1300 nm single photon emission of a metamorphic InAs Quantum Dot

G. Muñoz-Matutano[1-2*], D. Barrera[1], C.R. Fernández-Pousa[3], R. Chulia-Jordan[2], L. Seravalli[4], G. Trevisi[4], P. Frigeri[4], S. Sales[1], J. Martínez-Pastor[2*].

[1]ITEAM Research Institute, Universidad Politécnica de Valencia, C/Camino de Vera s/n, E-46022 Valencia, Spain

[2]Instituto de Ciencia de los Materiales, Universitat de València, PO Box 22085, E-46071 Valencia, Spain

[3]Departamento de Ingeniería de Comunicaciones, Universidad Miguel Hernández, Avenida Universidad s/n, E-03202 Elche, Spain

[4]CNR-IMEM Institute, Parco delle Scienze 37a, I-43100 Parma, Italy

[*]Corresponding author

Experimental Set-Up

Figure S1 shows the experimental set-up for μ-PL and μ-TRPL. The excitation scheme are similar than the photon correlation experiment described in figure 1 of the paper. For μ-TRPL wavelength the filtering is made by using a Fiber Bragg Grating (FBG), but μ-PL spectra were acquired by using standard monochromator filtering, as we are able to tune only about 8 nm using FBG filtering. We used a Id230 InGaAs Avalache Photodiode from IdQuantique as fast detector for μ-TRPL, which is correlated with the external triggering of the laser pulse. In μ-PL

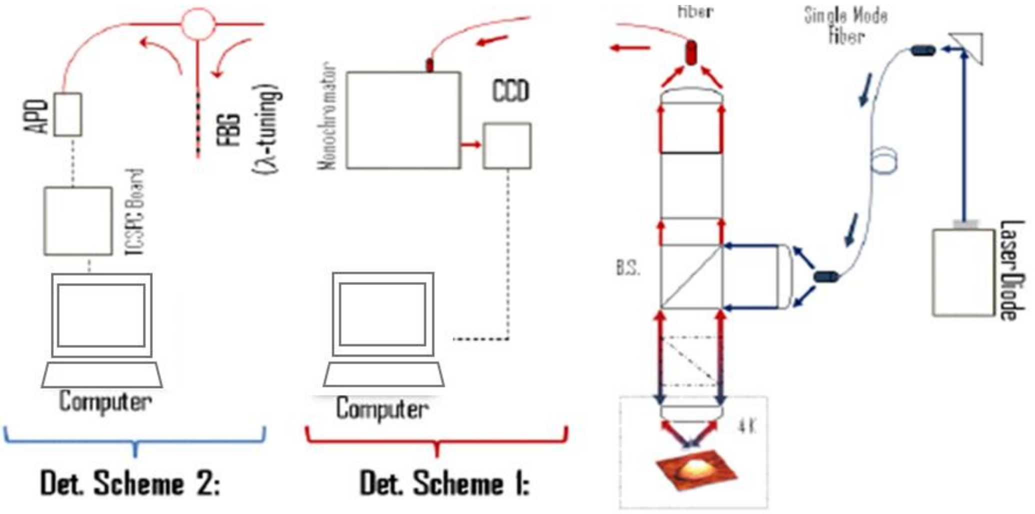

Figure S1.Experimental Set-Up for micro-Photoluminescence (μ-PL) and Time Resolved micro-Photoluminescence (μ-TRPL). Detection scheme 1 is used in the μ-PL arrangement, while detection scheme 2 is used for μ-TRPL measurement.

spectrum analysis we used an InGaAs Photodector Array from Andor Technology Ltd attached to a 0.5 m monochromator.

Time Resolved Photoluminescence spectroscopy:

The μ-PL transients in figure 2.b of the paper were recorded under 780 nm pulsed excitation. We used this excitation wavelength because 940 nm pulsed emission with our laser is not optimal, and we lost lot of laser intensity. μ-PL transients were fitted through the following equation, as previously reported [1]:

$$I(t) = I_0 + \frac{1}{w}\sqrt{\frac{\pi}{2}}exp\left(-\frac{2t^2}{w^2}\right) \otimes \left(A\left[exp\left(-\frac{t}{\tau_d}\right) - exp\left(-\frac{t}{\tau_r}\right)\right]H(t)\right) \qquad [S.1]$$

This expression is composed of uncorrelated, constant, noise background I_0 and the convolution ($\otimes$) of the system APD response (Gaussian profile with a linewidth w = 0.38 ns) with a single exponential rise-decay function (expression between parentheses) with time constants τ_r (rise time) and τ_d (decay time). The initiation of the excitation, conventionally set to t = 0, is represented by the Heaviside function, $H(t)$, which is zero for $t<0$ and one for $t>0$.

Power dependent μ-PL spectra using 780 nm cw excitation:

Figure S.2 shows power dependent μ-PL evolution under laser excitation at 780 nm (1,59 eV), i.e. above the GaAs barrier. Under this excitation condition neutral exciton complexes are more intense than the case of 940 nm excitation scheme, described in figure 2 of the main text. As it is shown in figure S.2, neutral biexciton transition (XX^0) dominates over positive charged biexciton (XX^{+1}). At the same time, neutral exciton (X^0) is more intense when exciting at 780 nm, dominating over all transitions from low to high power excitation range. The same trend is observed when comparing positive trion (X^{+1}) and positive quarton (X^{+2}) transitions. X^{+1} has similar intensity or dominates over X^{+2} along the whole excitation range. However, in figure 2 of the main text, X^{+2} dominates over X^{+1} at all excitation powers. A similar trend is observed when analyzing the negative trion recombination (X^{-1}). Under 780 nm excitation, X^{-1} transition is more intense than the case of 940 nm excitation.

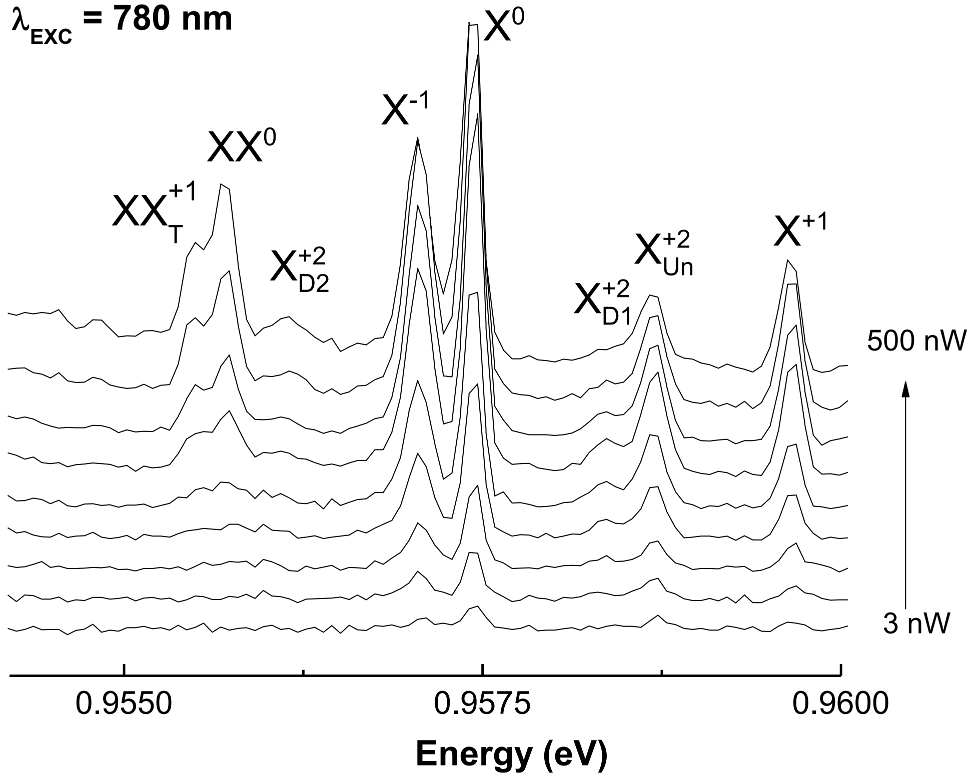

Figure S2. Power dependent micro-Photoluminescence spectra from the same single metamorphic InAs SAQD in figure 2, but using laser excitation at 780 nm (above the GaAs barrier).

All of these features are compatible with a lower injection rate of positive carriers under above GaAs barrier excitation (780 nm) than the case of nearly resonant excitation with the InAs Heavy Hole Wetting Layer state. We previously found a similar finding in smaller self-assembled QDs ([1], but also in first samples of big-size self-assembled QDs grown on metamorphic substrates [2], where a switching behavior between negative and positive carrier feeding is observed under different excitation conditions: above GaAs barrier or resonant with the residual acceptor impurity levels. The dynamical carrier feeding when using non-resonant excitation of QDs was a common feature previously investigated by many authors [3], [4], being associated to the presence of ionized acceptors and donor impurities in the GaAs barrier. This charge feeding helps to confirm our excitonic labelling in figure 2, as discussed in the paper. Figure S.2 shows a second effect: XX^0 and XX^{+1} transitions present broader linewidths under 780 nm excitation (≈210 μeV) than those measured at 940 nm (≈ 160 μeV). The linewidth broadening is attributed to the presence of a dynamical charge feed near the QD [5], [6], associated

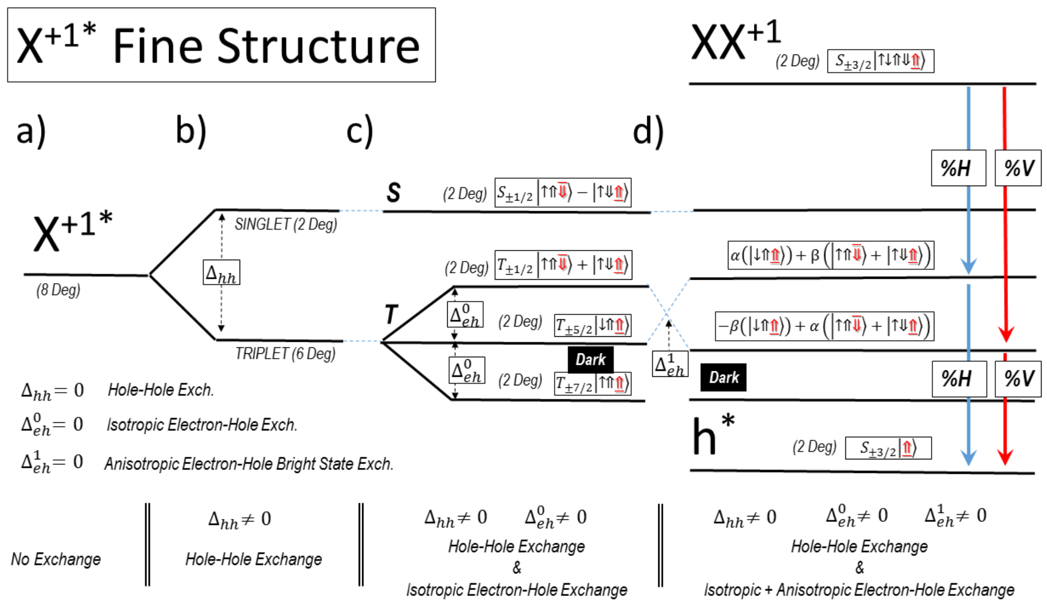

Figure S.3. State diagram of Hot Positive Trion (X^{+1*}), consisting of single electron in S shell ($\uparrow$ *or* $\downarrow$), single hole in S shell ($\Uparrow$ *or* $\Downarrow$) and single hole in P shell ($\Uparrow$ *or* $\Downarrow$). a) Eight fold degenerated state, without any exchange term under consideration. b) Singlet and triplet state due to the inclusion of the hole-hole exchange term (Δ_{hh}). c) Triplet degeneracy breaking by the inclusion of the isotropic electron-hole exchange term (Δ_{eh}^{0}). d) Mixing of triplet bright states by the inclusion of the anisotropic electron-hole exchange term (Δ_{eh}^{1}). It is represented cascade recombination from single positive charged biexciton (XX^{+1}) to the triplet state of X^{+1*}(labelled in the text as XX_T^{+1} transition), and from the triplet state X^{+1*} to single hole at P sate (h^*) (labelled in the text as X_T^{+1*} transition). The mixing effect of the Δ_{eh}^{1} exchange term produces partially linearly polarized optical transitions in the cascade recombination, described by blue (%H = partially horizontal) and red (%V = partially vertical) arrows.

to the ionization and neutralization of surrounding impurities. This charge fluctuation originates a spectral diffusion (SD) process that gives rise to the observed linewidth broadening. When using low excitation energy (i.e., 980 nm), the ionized acceptor band is not excited and hence the number of fluctuating dynamical charged states decreases and SD is weaker. On the other hand, SD is more pronounced in big QDs [5], due to the random motion of surface charges at the vacuum-semiconductor interface.

Positive Hot Trion state diagram:

As it has been noticed above, X^{+1*} is composed by one electron in the S level, one hole in S level and one extra hole in the P level. Since all energy levels of the QD are occupied here by only one particle, there are 2^3 different spin configurations. The presence of the resident charge changes the total spin of the system from integer to half integer value, and as a consequence of the Kramers theorem, all eigenstates of the trion are doubly degenerated in the absence of external time reversal breaking perturbation. Figure S.3-

a does not include any exchange term, and the state is eight fold degenerated. Exchange interaction between identical particles is much stronger than the one between different carriers. In this sense, hole-hole exchange interaction (Δ_{hh}) dominates over electron-hole exchange terms. Figure S.3-b shows the breaking of the eight fold degeneracy by hole-hole exchange interaction, splitting the positive hot trion into a singlet state with $J = 0$; $J_Z = 0$, and a triplet state with $J = 3; J_Z = 0; \pm 3$. However, experimental evidences based on the analysis of the fine structure of negative and positive quarton point out, that the Δ_{hh} exchange term breaks the degeneracy of the triplet state into a singlet state with $J_Z = 0$ and a doublet with $J_Z = \pm 3$ [7]. However, in any case, the degeneracy is broken by electron and hole exchange interaction. The electron and hole exchange interaction is composed by an isotropic term (Δ_{eh}^{0}) plus bright and dark anisotropic terms ($\Delta_{eh}^{1}, \Delta_{eh}^{2}$). The first one, Δ_{eh}^{0}, can be considered smaller in magnitude than the exchange term, Δ_{hh}, and hence it can be treated as an interaction of the electron spin with the total angular momentum of holes, J. On the other hand, Figure S.3-c illustrates the splitting of the sixfold degenerate triplet state into three doublets with $F_z = s + J_z = \pm 1/2, \pm 5/2, \pm 7/2$ by means of the isotropic electron-hole exchange term, where the last doublet state ($F_z = \pm 7/2$) is not optically active (Dark state). Finally, Figure S.3-d summarizes the final structure of the positive hot trion, under Δ_{hh}, Δ_{eh}^{0}, Δ_{eh}^{1} exchange interaction terms. The anisotropic term of the exchange ($\Delta_{eh}^{1}\ and\ \Delta_{eh}^{2}$) mixes these three doublets: Δ_{eh}^{1} gives rise to the mixing between $J_Z = \pm 1/2$ and $J_Z = \pm 5/2$ whereas Δ_{eh}^{2} to the mixing between $J_Z = \pm 5/2$ and $J_Z = \pm 7/2$), even if here we have considered that $\Delta_{eh}^{2}/\Delta_{eh}^{0} \ll 1$ and hence the mixing between $J_Z = \pm 5/2$ and $J_Z = \pm 7/2$ states would remain quite small [9].

From the basis of the above explained configuration for the positive hot trion state one is able to describe the polarization of the allowed biexciton recombination into a positive hot trion, and the subsequent optical transition towards the single excited hole (J^*). Following similar arguments described in ref [9], it is possible to develop the angular momentum selection rules including Δ_{hh}, Δ_{eh}^{0} exchange terms that produce circular polarization emission between XX^{+1} and X^{+1*} and the opposite direction circular polarization between X^{1+*} and J^*. However, when the anisotropic exchange term (Δ_{eh}^{1}) is taken into account, the mixing of triplet states generates a partially linear polarized

transitions (elliptical polarization), which, depending on the strength of the mixing, could reproduce strong linear polarization transitions H and V (labelled as %H and %V), as illustrated in figure S.2-d: transitions X^{+1} - X^{+1*} and X^{1+*} - J^{*} appearing when included all relevant hole-hole and electron-hole exchange terms. Therefore, we assigned the polarization dependent splitting observed in figure 3.b and 3.c in the paper to the FSS of X^{+1*} triplet state.

References

[1] G. Muñoz-Matutano, D. Rivas, A. Ricchiuti, D. Barrera, C. FernándezPousa, J. Martínez-Pastor, L. Seravalli, G. Trevisi, P. Frigeri y S. Sales. *Nanotechnology* **25**, 035204 (2014).

[2] G. Muñoz-Matutano, B. Alén, J. Martínez-Pastor, L. Seravalli, P. Frigeri y S. Franchi. *Nanotechnology* **19**, 145711 (2008).

[3] L. Seravalli, G. Trevisi, P. Frigeri, D. Rivas, G. Muñoz-Matutano, I. Suárez, B. Alén, J. Canet-Ferrer y J. Martínez-Pastor. *Appl. Phys. Lett.* **98**, 173112 (2011).

[4] E. S. Moskalenko, K. F. Karlsson, P. O. Holtz, B. Monemar, W. V. Schoenfeld, J. M. Garcia y P. M. Pretroff. *Phys Rev B* **66**, 195332 (2002).

[5] W. H. Chang, H. S. Chang, W. Y. Chen, T. M. Hsu, T. P. Hsieh, J. I. Chyi y N. T. Yeh. *Phys Rev B* **72**, 233302 (2005).

[6] N. Ha, T. Mano, Y.-L. Chou, Y.-N. Wu, S.-J. Cheng, J. Bocquel, P. M. Koenraad, A. Ohtake, Y. Sakuma, K. Sakoda y T. Kuroda. *Phys Rev B* **92**, 075306 (2015).

[7] M. Abbarchi, F. Troiani, C. Mastrandrea, G. Goldoni, T. Kuroda, T. Mano, K. Sakoda, N. Koguchi, S. Sanguinetti, A. Vinattieri y M. Gurioli. *Appl Phys Lett* **93**, 162101 (2008).

[8] M. Ediger, G. Bester, B. Gerardot, A. Badolato, P. Petroff, K. Karrai, A. Zunger y R. Warburton. *Phys. Rev. Lett.* **98**, 036808 (2007).

[9] I. Akimov, K. Kavokin, A. Hundt y F. Henneberger. *Phys. Rev. B* **71**, 075326 (2005).
